# Pyorrhœa Alveolaris

**Published:** 1904-03

**Authors:** Frank R. Mayer

**Affiliations:** Worcester, Mass.


### PYORRHŒA ALVEOLARIS.[1]

BY FRANK R. MAYER, D.D.S., WORCESTER, MASS.

HAVING been requested to present this paper to you, which I read at the Central District Dental Society, I immediately felt that the paper in its original entirety was too incomplete, the subject having been handled from a point starting from the supposed and almost unmistakable diagnosis familiar to the average dentist also treating the subject, omitting much that might have been said, because of a previous understanding of the Society that the reading of papers should require but the short space of five minutes.

Hence you can see that my paper, unless exceeding the allotted five minutes, must be incomplete.

I have treated this subject pathologically from what many consider its secondary cause,—namely, salivary calculus, having ignored the primary causes, constitutional disturbances, blood impoverishments, and serumal deposits, which I fear many like myself are uncertain whether to consider a prominent manifestation worthy our first consideration, or, to take its place, a possible cause which we in our uncertainty cannot lose sight of. It is left to the dentist, whose profession entitles him to the highest respect, to deserve such respect in a befitting manner, and, through a scientific research, to place upon a plane of unmistakable certainty the true cause of this devastating disease, that we may treat the disease unhampered by mistrust or any unsatisfactory previous disposition.

A cure must be effected from our starting-point, which is a proper knowledge of the probable cause, whether hereditary or acquired, from which point we can effect a cure. Often we find this lesion flourishing in patients without a history of rheumatic diathesis, blood impoverishments, or other abnormalities; we have found it in patients presenting characteristics dissimilar in many ways; how then can we discover it, unless by the chemical or analytical treatment of found conditions. There must be but one primary cause for this disease. I have treated the subject in my original paper mostly from a mechanical point of view, leaving the pathological causes which lead to this disease to those men who have scientifically endeavored to unearth the secret. I will now r ad the original paper as read at the infant society.

I feel that I owe you all an apology in attempting to handle a subject of which there has been so much written, yet one not altogether understood and, until recent years, not satisfactorily treated.

Pyorrhœa alveolaris, I consider, ranks second to none in stubborn defiance and resistance to the skill of the persistent dentist. Perhaps at this point it would be well to revert to history and acquaint ourselves with the origin of this disease, the pathological diagnosis of which we owe to Dr. Riggs; also its name,—Riggs's

---

[1] Read before the Massachusetts Dental Society, Boston, Mass., June 3 and 4, 1903.

disease. Resorting to a more remote past, we find evidence that the disease was treated with more or less success years before the lesion presented itself to Dr. Riggs. I can see no reason why the disease should not bear the name of Riggs, not that he was the first to treat it, but because he made it a distinct lesion, classifying it as necrosed alveolar process. So we understand that the disease called Riggs's disease is a distinctive form, like others, named after persons who first described their characteristics, as Bright's disease, etc.

The disease has been treated with varying success since 1746. The treatment was then divided into two classes,—first, surgical, and second, medical. Among the surgical methods is the extracting of the teeth, thus ending the infection of the disease to other healthy dental articulations. This method was soon considered to be a too severe loss, and other surgical methods followed quickly. Among these was a V-shaped incision corresponding to the shape of the tooth, its summit directed towards the apex of the root, forming a triangular flap; this flap was then removed and a cautery passed over the basic portion, and was then allowed to heal naturally.

American dentists modified this treatment by lancing from the apex towards the neck of the tooth, then cauterizing the under surface of the flap with nitrate of silver and carbolic acid.

Dr. Riggs's surgical method was to scrape the surface of the root of the affected tooth to remove all salivary calculi, and also removing any necrosed bone, then continuing with the cauterizing. Even extreme methods, as replantation, were resorted to where there was elongation of the incisor teeth.

Many medical methods have been used to conquer the disease, such as the use of bichloride solutions, aromatic sulphuric acid, iodide of zinc, iodoform, ether, aristol, caustic potash, carbolic acid, peroxide of hydrogen, nitrate of silver, etc.

The most important therapeutic consideration in this disease is the local treatment, yet we cannot lose sight of the constitutional treatment. The disease generally begins with gingival inflammation, then periostitis or inflammation of the periosteum; when the attack has destroyed the outer crust of the bone the osseous tissue is open for infection.

The first step towards the treatment of this disease is the removal of tartar, also the affected alveolus. The removing of the tartar at first thought seems easy, but upon closer observation we find that what we supposed we had dislodged remains to partly undo what would otherwise have proved a successful step towards complete recovery.

We have the visible and invisible tartar. It is quite an easy matter to remove the visible, but to thoroughly remove the tartar which runs oftentimes to the extreme end of the root is a problem that can only be solved by the dexterous use of appropriate instruments peculiarly adapted for this purpose, such as the several forms of scalers. In my own practice I have two instruments or scalers, one whose cutting edge cuts towards the operator or neck of the tooth, using a drawing motion; the other quite the reverse, its cutting edge acquiring a direct pushing motion towards the apex of the root; this latter instrument, because of its thin edge, obviates the otherwise painful operation of removing the tartar at the extreme end of the root.

After we feel confident of the complete removal of this tartar we often find a slight nodosity, which must be removed or scraped until smooth and well polished, as the least particle of life forms a new alveolar infection. This point I consider the pivot upon which the dentist meets with success or failure.

In spite of the most favorable instruments, the operation is often attended with unbearable pain. I have used with success a local application of cocaine, which, when applied to gum corresponding to and opposite the apex of the root, has rendered that tissue insensible to pain, consequently satisfactory to both operator and patient. Peroxide of hydrogen can be used with success because of its peculiar properties, forcing any particle of loose calculus to the surface, which can then be washed out.

We often find the disease has progressed to the stage where there has been a severe loss or disintegration of the bone, first attacking the periosteum, then the inner structure of the bone, resulting in the death of that organ.

After the use of peroxide of hydrogen I have generally used sulphuric acid to cauterize each gum pocket, but have lately found this to be too strong for the healthy granulation of surrounding tissue, aside from the danger of exposing the dentinal fibrils, resulting in extreme sensitiveness.

You are probably aware of a preparation that has recently appeared for dental use which has been adopted with success in some instances. It is named "Glyco-thymoline." I believe that any antiseptic for oral use should be alkaline in reaction, as the least acidity is dangerous to the teeth and connective tissues. The secretions of the mouth normally are found to be alkaline, and when diseased they become acid; so we need a strong alkaline reaction to produce a normal condition of the secretions.

We must have an antiseptic that is strongly alkaline, one that will not coagulate albumin; it should be a deodorant, non-irritating, and, in fact, should correspond to the natural healthy constituents of normal blood and mouth secretions.

In several patients I have found the following efficacious in arresting the rapid formation of tartar:

℞ Citrate of lithia, gr. x;

Glyco-thymoline, ℥iv.

Sig.—Inject deep into the pus-pockets, this treatment to be followed every few days until there are signs of complete restoration to a healthy condition.

Massaging the gums often increases the capillary circulation, the lack of which is one of the principal causes of this disease. We often meet the disease advanced to the stage when the teeth have become loosened. I have found ligation, in the not too far advanced stage, satisfactory in lessening the danger of increasing the surrounding inflammation, but when the gums have loosened to such an extent as to entirely fail to support the teeth I use a number of bands, either of gold or other suitable metal, made to tightly fit the necks of the teeth well up towards the biting edge. After placing these bands in position I take an impression, removing bands in same, soldering bands together in relative positions, and then replacing with use of cement if found necessary; then proceed with treatment heretofore described.

I might speak of several cases that have come under my observation and for treatment, but would make the paper too long, so will desist.

